# Optimized Encapsulation of the FLAP/PGES-1 Inhibitor BRP-187 in PVA-Stabilized PLGA Nanoparticles Using Microfluidics

**DOI:** 10.3390/polym12112751

**Published:** 2020-11-20

**Authors:** Mira Behnke, Antje Vollrath, Lea Klepsch, Baerbel Beringer-Siemers, Steffi Stumpf, Justyna A. Czaplewska, Stephanie Hoeppener, Oliver Werz, Ulrich S. Schubert

**Affiliations:** 1 Laboratory of Organic and Macromolecular Chemistry (IOMC), Friedrich Schiller University Jena, Humboldtstraße 10, 07743 Jena, Germany; mira.behnke@uni-jena.de (M.B.); antje.vollrath@uni-jena.de (A.V.); lea.klepsch@uni-jena.de (L.K.); baerbel.beringer-siemers@uni-jena.de (B.B.-S.); steffi.stumpf@uni-jena.de (S.S.); justyna.czaplewska@uni-jena.de (J.A.C.); s.hoeppener@uni-jena.de (S.H.); 2 Jena Center for Soft Matter (JCSM), Friedrich Schiller University Jena, Philosophenweg 7, 07743 Jena, Germany; oliver.werz@uni-jena.de; 3 Department of Pharmaceutical/Medicinal Chemistry, Institute of Pharmacy, Friedrich Schiller University Jena, Philosophenweg 14, 07743 Jena, Germany

**Keywords:** BRP-187, FLAP inhibitor, mPGES-1 inhibitor, PLGA nanoparticles, microfluidic, staggered herringbone mixer, drug encapsulation, nanoparticle formulation

## Abstract

The dual inhibitor of the 5-lipoxygenase-activating protein (FLAP) and the microsomal prostaglandin E_2_ synthase-1 (mPGES-1), named BRP-187, represents a promising drug candidate due to its improved anti-inflammatory efficacy along with potentially reduced side effects in comparison to non-steroidal anti-inflammatory drugs (NSAIDs). However, BRP-187 is an acidic lipophilic drug and reveals only poor water solubility along with a strong tendency for plasma protein binding. Therefore, encapsulation in polymeric nanoparticles is a promising approach to enable its therapeutic use. With the aim to optimize the encapsulation of BRP-187 into poly(lactic-*co*-glycolic acid) (PLGA) nanoparticles, a single-phase herringbone microfluidic mixer was used for the particle preparation. Various formulation parameters, such as total flow rates, flow rate ratio, the concentration of the poly(vinyl alcohol) (PVA) as a surfactant, initial polymer concentration, as well as presence of a co-solvent on the final particle size distribution and drug loading, were screened for best particle characteristics and highest drug loading capacities. While the size of the particles remained in the targeted region between 121 and 259 nm with low polydispersities (0.05 to 0.2), large differences were found in the BRP-187 loading capacities (LC = 0.5 to 7.29%) and drug crystal formation during the various formulations.

## 1. Introduction

Nowadays, the detection and treatment of human diseases rely more and more on nanotechnology, more precisely on nanomedicine. Nanomedicine describes the use of nanoscale carriers containing either therapeutic agents (e.g., drugs, genetic material) or diagnostic entities (e.g., dyes, radioactive elements) or even both (theranostic carriers) [1,2,3]. In this field, besides lipid-based nanocarriers, polymer-based nanoparticles (NPs) play a major role [3,4]. Due to the wide range of monomers from which the polymers can be constructed and the resulting multifunctional architectures and options for post-modification, polymer-based NPs are highly potential candidates as drug delivery vehicles for the treatment of inflammation, cancer, and other diseases [3,4]. One of the most frequently applied polymers for drug encapsulation due to its beneficial properties, such as high biocompatibility and its excellent degradation into H_2_O and CO_2_, is poly(lactic-*co*-glycolic acid) (PLGA) [5,6,7]. The PLGA NPs’ physicochemical properties can be fine-tuned and are mainly influenced by the used PLGA and formulation technique [6]. With a variation of the polymer molar mass and the ratio of lactic acid (LA) and glycolic acid (GA) units, the degradation time, crystallinity, and hydrophobicity of the NPs can be modified [8].

As recently reported by our group, PLGA can be also used for the encapsulation of the anti-inflammatory drug BRP-187 (4-(4-chlorophenyl)-5-(4-(quinoline-2-ylmethoxy)phenyl)isoxazol-3-carboxylic acid) [9]. This drug was discovered in 2016 within a class of dual inhibitors that are able to reduce the formation of pro-inflammatory mediators without affecting the production of pro-resolving mediators for improved therapy [10,11,12]. In detail, these inhibitors dually block the microsomal prostaglandin E_2_ synthase-1 (mPGES-1) as well as 5-lipoxygenase (LO) or 5-LO-activating protein (FLAP) and suppress efficiently prostaglandin E_2_ (PGE_2_) and leukotriene levels without the unfavorable side effects of non-steroidal anti-inflammatory drugs (NSAIDs) [10,11,13,14]. However, BRP-187 is an acidic lipophilic drug and reveals only poor water solubility along with a strong tendency for plasma protein binding and relies on the entrapment in NPs [9]. For a first in vitro evaluation of BRP-187-loaded PLGA NPs (PLGA(BRP-187)), a batch co-nanoprecipitation process was applied, and particles with 130 to 168 nm in size and loading capacities (LC) of up to 2.2% to 2.5% were obtained [9]. Improved stability, bioavailability, and efficiency of the BRP-187-loaded NPs compared to the free compound in suppressing 5-LO product formation and PGE_2_ biosynthesis in intact cells were observed and underlined the promising perspective for the use of this substance/class of substances in the near future [9]. In view of upcoming in vivo studies or clinical applications, the formulation still lacks control and precision, particularly in terms of higher LC values and scale-up possibilities [15,16,17]. Thus, in order to eliminate the drawback of the conventional bulk nanoprecipitation method [15] and to increase the final drug loading capacity while the size and dispersity of the particles remain similar, the preparation of the PLGA(BRP-187) NPs was transferred from batch nanoprecipitation to a microfluidic approach in the presented study. 

Microfluidics as a contemporary technology offers a multitude of application possibilities by manipulating fluids in microchannels and is becoming increasingly important not only in point-of-care testing, analysis, biotechnology, and chemistry but above all for the formulation of NP-based pharmaceuticals and personalized medicine [18,19]. Nowadays, reams of microfluidic chips are available with all kinds of geometries that control the fluids and the mixing of fluids either actively or in a passive manner [20]. Active micromixers use an external driving force, e.g., magnetic stirring to enable the mixing, whereas passive micromixers use certain channel geometries to increase the interfacial contact area between the mixing species, e.g., diffusion mixers with very long channels or special substructures, such as staggered herringbone mixers to induce chaotic flows [20]. The chaotic mixing in a staggered herringbone mixer is very fast and efficient and is, therefore, often used for the manufacturing of nanocarriers, such as lipid [21] and polymeric nanoparticles (NPs) [22], amongst others [23]. In particular, for drug delivery applications, the rapid mixing during the preparation of NPs is of vital importance since the formation of polymeric NPs typically occurs in three steps: (i) nucleation of NPs (these nuclei are composed of multiple polymeric monomers), (ii) growth phase (additional monomers are bound by a diffusion-limited process), and (iii) equilibrium (the NPs grow until they are kinetically locked by a polymer corona formed on their surface after a specific aggregation time scale) [22,24]. In a classic batch formulation, these phases proceed simultaneously as the mixing process is barely controlled, and no separation takes place during particle growth from the aggregation process. However, in microfluidics, the first two steps (nucleation and growth phase) of NP formation may be considered as a function of the distance from the position where the solution is mixed, and this results in a good control over NPs size, size distribution, as well as their morphology [22]. The NPs properties can be thereby influenced not only by the chip properties, e.g., the aforementioned channel dimensions and structure, but also by the flow velocities and flow rate ratios applied [21,23,25,26]. Ultimately, an improvement in reproducibility and lower batch to batch, and also inter-batch, variance compared to conventional batch processes can be achieved with microfluidics not only in nano- and microscale but also for scale-up production [17,27]. Moreover, increased drug loads are described to be obtainable in combination with microfluidic approaches [27,28], which are a crucial point since, for many drug delivery systems, sufficiently high LC with good particle characteristics remain challenging. It is estimated that 40% of the currently marketed drugs and around 90% of molecules in the development pipeline exhibit poor water solubility and rely on efficient encapsulation into nanocarriers to overcome the low bioavailability, safety, efficacy, and patient compliance [29]. Thus, it is of central interest to constantly optimize the encapsulation of drugs into NPs—not only in terms of the best physicochemical properties of the particles but also in terms of a sufficient drug loading [30].

To this end, a staggered herringbone mixer chip from microfluidic ChipShop, Jena, Germany, was used that could be easily connected to syringes (with or without the usage of syringe pumps) and is very versatile for application in basically every lab around the world, but it was not established for polymeric NPs formulations up to now [31,32,33,34,35]. A formulation screening of the PLGA(BRP-187) NPs was performed with this mixer chip, whereby important parameters, such as flow velocities, flow rate ratios, solvents, initial concentration of PLGA, BRP-187, as well as of the surfactant poly(vinyl alcohol) (PVA), were varied to determine the best conditions for increased drug loading and stability [21,23,25,26,36,37]. Moreover, different purification protocols were established. All obtained particles were analyzed using dynamic light scattering and scanning electron microscopy measurements, as well as UV-Vis spectroscopy, for the determination of the final particle size, distribution, shape, charge, and stability, as well as LC and encapsulation efficiency (EE).

## 2. Methods

### 2.1. Materials

The COOH terminated poly(lactic-*co*-glycolic acid) (PLGA) copolymer (Resomer RG 502 H, Evonik, Essen, Germany) with a ratio of LA:GA of 50:50 and a molar mass of 7–17 kDa was chosen for formulation. The inhibitor BRP-187 was synthesized according to a published procedure (Figure SI 1 in the Supplementary Materials) [12]. The solvents—acetone (Ac), ethyl acetate (EtOAc), tetrahydrofuran (THF), acetonitrile (ACN), and dimethyl sulfoxide (DMSO), as well as the partially hydrolyzed PVA (Mowiol 4-88, DH 88.0%), were purchased from Sigma Aldrich, Steinheim, Germany. Lugol solution was purchased from Carl Roth, Karlsruhe, Germany.

### 2.2. Nanoparticle Formulation

The formulation of the NPs was carried out by nanoprecipitation using a microfluidic technique. Therefore, a single-phase herringbone mixer chip from microfluidic ChipShop, Jena, Germany, was utilized. It consisted of two inlet channels with 200 µm (depth) × 300 µm (width), a channel mixer (staggered herringbone) of 600 µm width, and a channel width outlet of 600 µm. The chip was connected to two syringes via polytetrafluoroethylene (PTFE) tubing (ID: 0.5 mm, OD: 1 mm, microfluidic ChipShop, Jena, Germany), which were automatically controlled by two low-pressure syringe pumps (neMESYS 290N, Cetoni, Korbußen, Germany) (Figure SI 2). To study the influence of the flow rates, PVA concentration, solvent/co-solvent, initial polymer concentration, and initial drug feed, different protocols were used. In total, six different flow rates (0.4:0.1 mL min^−1^, 2.0:0.5 mL min^−1^, 4.0:0.5 mL min^−1^, 6.0:0.5 mL min^−1^, 8.0:0.5 mL min^−1^, and 8.0:2.0 mL min^−1^), three different PVA concentrations (0.3% (*w*/*w*), 1.0% (*w*/*w*), and 3.0% (*w*/*w*)), four different solvents/co-solvents (acetone, EtOAc, THF, ACN), three different polymer concentrations (5 mL min^−1^, 15 mL min^−1^, and 25 mL min^−1^), and three different BRP-187 concentrations (3%, 5%, and 10%) were tested. The polymer was dissolved in the respective solvent, and the inhibitor BRP-187 was added to this solution and filled into the first syringe. The second syringe was filled with water containing the PVA surfactant. Before starting the formulation, a short rinsing (pre-wetting step) of the chip with the prepared water/PVA solution was performed. The formulation was collected in a glass vial, stirred at room temperature, and evaporated for 2 to 4 h.

### 2.3. Nanoparticle Purification

After the formulation and evaporation of the solvent, the particles were washed once using a 5804 R centrifuge (Eppendorf, Hamburg, Germany) with 11,000 rpm for 90 min at 20 °C. The supernatant was discarded, and 2.5 mL of water was added to resuspend the particles. For that reason, the particles were vortexed for around 15 s and afterward sonicated for 30 min in an ultrasound bath. After that, the particles were stored at 4 °C overnight for full resuspension. For further studies, five aliquots of 200 µL were freeze-dried the next day (Lyophilizer Christ Alpha 2–4 LD plus, Osterode, Germany). The NPs mass was determined by using an MYA 11.4Y microbalance (Radwag Waagen, Radom, Polen).

### 2.4. Dynamic Light Scattering

The characterization of the purified particles in terms of size, polydispersity, and zeta-potential was performed by DLS measurements using a Zetasizer Nano ZS with a laser wavelength of λ  =  633 nm (Malvern Panalytical GmbH, Malvern, UK). Each measurement of the size and polydispersity index (PDI) was implemented in UV cuvettes consisting of polystyrene (Brand GmbH + Co KG, Wertheim, Germany) with five runs of 30 s after an equilibration time of 30 s, while the backscatter angle was set at 173°. To determine the NPs zeta-potential, 10 µL of the suspension was diluted with 1 mL water and measured three times at 25 °C. The size distribution by intensity was used to gather the hydrodynamic diameter (d_H_) of the NPs.

### 2.5. UV-Vis Spectroscopy

The encapsulation efficiency (EE) and loading capacity (LC) were evaluated by using the Infinite M200 Pro plate reader (Tecan Group, Männedorf, Switzerland). Therefore, three lyophilized samples were dissolved in 200 µL DMSO, and 100 µL of this solution was pipetted in a well of a Hellma Quartz flat-transparent plate with 96 wells. Undiluted and diluted samples (factor 2:1, 4:1, and 12:1) were measured at a wavelength of λ  =  316 nm with 3  ×  3 multiple reads per well and a well border of 2000 µm. For each measurement, a new calibration curve of the BRP-187 in a range of 250 to 0.244 µg mL^−1^ with R^2^  =  0.9982 to 1 was created (Figure SI 6).

$$\mathrm{LC}=\frac{mass\;of\;drug\;recovered}{mass\;of\;particle\;recovered}\;\times \;100$$

$$\mathrm{EE}=\frac{\mathrm{LC}}{\mathrm{LC}\;theoretical}\;\times \;100$$

### 2.6. PVA Assay

For the quantification of the PVA residue in the NP formulation, a PVA assay was performed via UV-Vis spectroscopy. Due to the complex formation of PVA with iodine of a Lugol solution, the absorption is measurable at λ  =  650 nm [38]. One freeze-dried aliquot was used and resuspended in 1 mL pure water. The 90 µL of the resuspended suspension was transferred into a VWR^®^ Tissue Culture 96 wells-F plate. This was repeated for each sample three times. Afterward, 20 μL of 1 M sodium hydroxide was added to each well, and the mixture was incubated for 15 min by using a BioShaker with 850 rpm at room temperature to degrade the particles via hydrolyzation of the NP matrix. Subsequently, 20 µL of a 1 M hydrochloric acid was added for neutralization. The complexation started after adding 60 µL 0.65 M boric acid and 10 µL Lugol solution. Next, the sample absorption was measured within 15 min with the plate reader. The associated calibration curve was set up analogously to the NP samples.

### 2.7. Scanning Electron Microscopy (SEM)

Another method for the investigation of NPs is scanning electron microscopy (SEM). For the imaging of the particles, a Sigma VP Field Emission Scanning Electron Microscope (Carl-Zeiss, Jena, Germany) with an Inlens detector and an accelerating voltage of 5 to 6 kV was used. Therefore, the samples were previously coated with a 4 nm platinum layer by using a CCU-010 HV sputter (Safematic, Zizers, Switzerland). For the evaluation of the NPs sizes, ImageJ was used.

## 3. Results and Discussion

**Figure 1:**
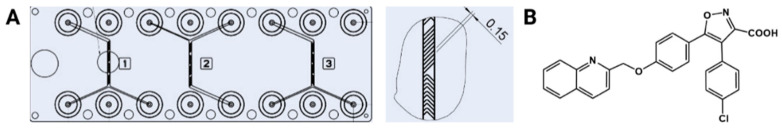
(**A**) Herringbone mixer chip (3 channels per chip, consecutively numbered 1–3, ChipShop, Jena, Germany) with two inlet channels with 200 µm (depth) × 300 µm (width), channel mixer (staggered herringbone) of 600 µm width, and a channel width outlet of 600 µm [31]. (**B**) Structure of the anti-inflammatory drug BRP-187 (4-(4-chlorophenyl)-5-(4-(quinoline-2-ylmethoxy)phenyl)isoxazol-3-carboxylic acid) [11].

Nanoprecipitation, as a formulation method for NPs, is impressive with its simplicity and efficiency [17] and was, therefore, applied as the first technique for the encapsulation of BRP-187 (Figure 1B) into PLGA particles [9]. However, the bulk procedure is often limited with respect to increasing the polymer concentration and drug loading without increasing the particle sizes and distributions as well as stability problems [9,15]. To overcome the lack of control and precision during particle formation by bulk preparation, the formulation of BRP-187 in PLGA particles was investigated within a herringbone staggered mixing chip (Figure 1A). The chip is made of Zeonor, a cyclo-olefin polymer, which has high transparency, high purity, and good fracture resistance. Zeonor is resistant to many solvents, such as DMSO, acetone, methanol, ethanol, and N,N-dimethylformamide. It is, therefore, widely used in medical and pharmaceutical applications. Using this chip offers the same advantages as nanoprecipitation (easy, fast, mild conditions), but the NP formation takes place in a microchannel with narrow and limited dimensions and, thus, happens rapidly and very efficiently [32,33,34]. The NPs properties can be influenced by the variation of crucial parameters, which can be divided into two categories: materials and settings. Materials include the properties of the polymer and drug, the characteristics of the surfactant, and the used polymer solvent. Within the settings, the concentration of the polymer, drug, and surfactant, as well as the flow rate velocities and flow rate ratios, are considered. On the basis of the already completed and positive study [9], the first set of experiments was started using acetone as a solvent for the PLGA polymer (c = 5 mg mL^−1^), an initial drug load of 3% BRP-187 (*w*/*w* PLGA), and an aqueous phase containing 0.3% of PVA as a surfactant. The total amount of PVA in each sample (Table SI 1 in the Supplementary Materials) was determined using the PVA assay described to calculate the actual loading capacities of the NPs. 

### 3.1. Variation of the Flow Rates

For the production of particles, flow rates in the range of 0.4 up to 8.0 mL min^−1^ were selected, whereby different flow rate ratios of 1:4 up to 1:16 for polymer phase to aqueous phase were chosen. Unloaded, as well as drug-loaded, particles were prepared with different flow rate ratios and velocities and subsequently investigated with DLS with respect to their final size distributions (P1–P9 Table SI 1). It was observed that independent from the presence of the drug, the same size range and similar PDI values were obtained at the respective flow rates (Figure SI 3A), which is beneficial as it shows that the drug had no negative influence at this feed concentration on the NP. SEM images of the samples confirmed the formation of monomodal and spherical particles (Figure SI 3B). 

**Figure 2:**
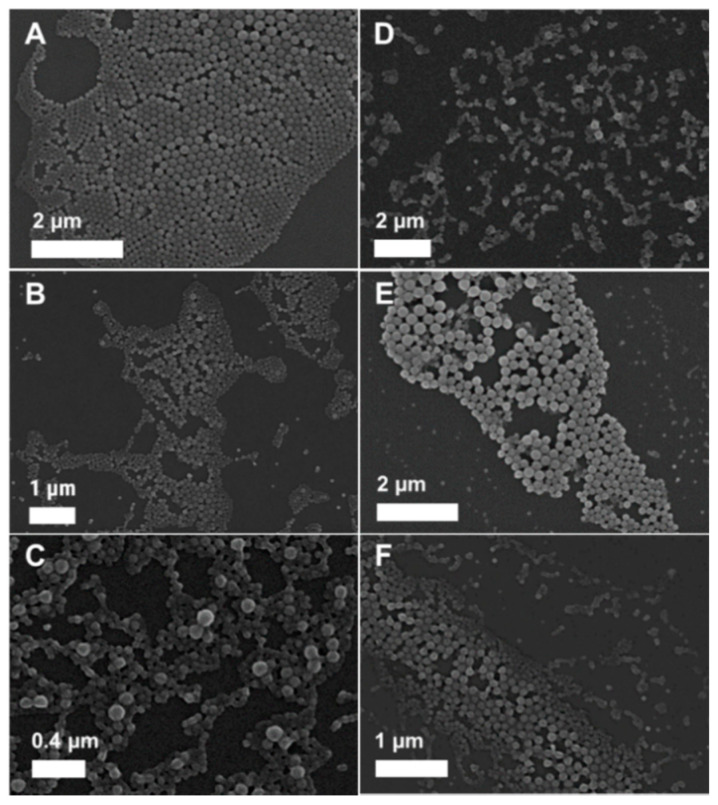
SEM images of PLGA(BRP-187) particles prepared with 5 mg mL^−1^ as polymer concentration and 3% BRP-187 (*w*/*w* PLGA) with the different flow rates (**A**–**F**); (**A**)—P4, (**B**)—P5, (**C**)—P6, (**D**)—P7, (**E**)—P8, and (**F**)—P9. PLGA, poly(lactic-*co*-glycolic acid).

Furthermore, it became obvious that with increasing velocities, the average particle size decreased from 212 to 121 nm, while the PDI value was increasing from 0.05 to 0.16, whereby it was particularly high for the highest flow rate ratio Q_w_:Q_PS_ = 8.0:0.5 (Figure SI 4A). This increase in the dispersity of the particles with increasing flow rates was also confirmed by SEM measurements (Figure 2A–F). The results are in good agreement with the literature, where it is also reported that the flow rate velocities and ratios significantly influence the final particle sizes and the distributions [39]. The faster the flow rate, the more rapidly the mixing occurs and, thus, the Reynold number increases, which means that the viscous frictional force becomes weaker compared to the inertial force. For the adaptation of the protocol to the later intended use, it should be considered that the particle size cannot be reduced indefinitely by increasing the speed as the size distribution increases at the same time. The best results in terms of PDI values were achieved for a moderate flow rate ratio of water to polymer Q_w:_Q_PS_ = 2.0:0.5 mL min^−1^, where PLGA(BRP-187) particles (P5) with sizes of 172 nm and very low PDI values of 0.05 as well as excellent reproducibility were obtained.

The monomodal particle distribution was also confirmed by SEM measurements (P5, Figure 2B), but the calculated average size from the SEM images (d_SEM_) was much smaller with d_SEM_ = 85 nm for the loaded and d_SEM_ = 88 nm for unloaded NPs (P2). For DLS analysis, the particles are measured in suspension in a hydrated state with a surrounding shell, which makes the particles larger [40]. On the contrary, SEM measures the particles in a dried state, whereby the particles often undergo shrinking during the drying process. Furthermore, the calculated values of SEM are based on the number distribution, whereby the calculated Z average values in DLS are based on the intensity distribution, which overrepresents larger particles. Thus, the observed difference in the average sizes was expected and is in agreement with previous findings [9,41,42]. All values calculated from the SEM images for particles prepared via different flow rates are listed in Table SI 1. After the characterization of the particles with respect to their size, shape, and surface charge, the LC and EE values were determined by UV-Vis measurements. Low LC values between 0.33% and 0.89% (Figure SI 4B) with corresponding EE values of up to 29% (Table SI 1) were determined. The low values reveal insufficient encapsulation in comparison to the original study utilizing bulk nanoprecipitation, which yielded LC values of 2.2% to 2.5% [9]. One reason for the low EE might be that not enough polymer molecules are available to adequately surround the drug and, therefore, the BRP-187 is precipitating already before it can be packed into the polymer, and thus, most of the drug is removed during the washing process.

### 3.2. Variation of the Solvent/Co-Solvent (Ratio)

**Figure 3:**
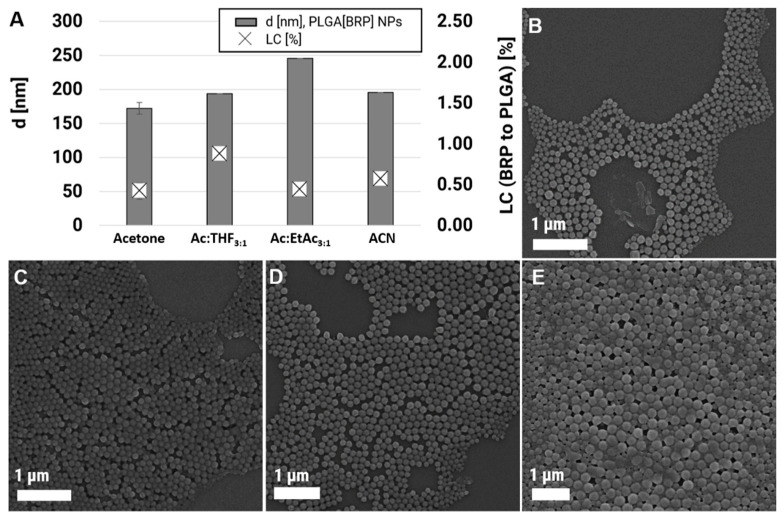
(**A**) Size and LC values. (**B**–**E**) SEM images of PLGA NPs formulated with Ac (P2, **B**), Ac:THF (P10, **C**), Ac:EtOAc (P11, **D**), as well as ACN (P12, **E**). LC, loading capacities; NPs, nanoparticles; Ac, acetone; THF, tetrahydrofuran; EtOAc, ethyl acetate; ACN, acetonitrile.

Since it is known that the solvents affect, for example, the particles size, polydispersity, as well as entrapment efficiency [43,44], different solvents, i.e., Ac, ACN, EtOAc, and THF, were investigated with regard to their suitability for the preparation of loaded PLGA(BRP) particles. Two aspects need to be considered as the most important parameters determining the NP properties: (i) the water miscibility of the solvents and (ii) the affinity of the polymer to the solvents [45]. It has been reported that with decreasing water miscibility of a solvent, the particle sizes are increasing [46,47]. Furthermore, it has been observed that a higher solvent-polymer affinity results in a larger supersaturation region at the liquid–liquid interface, which leads to larger particles and more non-uniform NP formation [46,47]. Good solvents for PLGA are usually Ac, EtOAc, THF, and chlorinated solvents, such as dichloromethane [44]. However, during the selection of the solvents, also special attention was paid to the durability of the chip since the chip material is made of Zeonor and is, therefore, sensitive to certain solvents. Ac and ACN remained as pure solvents of choice, and EtOAc, as well as THF, were only applicable to 25% as a mixture with acetone (Ac:EtOAc 75:25 and Ac:THF 75:25). Polymer, drug, as well as PVA concentrations, were kept constant for a better comparison of the final size, LC, and EE values. Besides, the flow rate was also fixed to a moderate velocity of 2.0:0.5 mL min^−1^ since with this flow rate, small but defined particles could be produced, and pressures within the chip systems were still low. The particle characteristics and SEM images of the formulations using different solvents are presented in Table 1 (P10–P12) and Figure 3. The formulation with co-solvents led to larger but more defined NPs, whereby the solvent mixture Ac:THF yielded NPs with sizes of 194 nm and the formulation with Ac:EtOAc particles with d = 246 nm. The application of ACN as solvent resulted only in slightly larger particles in the range of 200 nm (d = 196 nm) compared to acetone. These findings are in good agreement with the literature [46,47] since the water miscibility is declining in the following order: Ac > ACN > THF > EtOAc, whereas the particle sizes are increasing in the opposite direction [48,49,50]. Unfortunately, the LC values remained in the same low region as for pure acetone. The LC values were determined to be 0.45% for Ac:EtOAc and 0.59% for the solvent ACN and increased only slightly to LC = 0.89% for Ac:THF [51].

### 3.3. The Importance of the Initial Polymer Concentration

**Figure 4:**
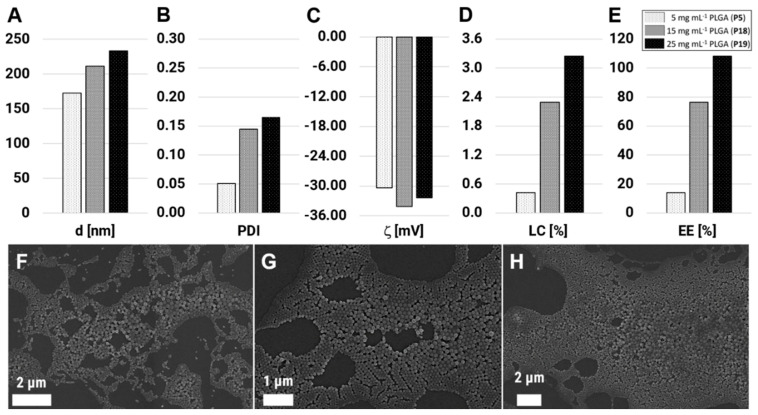
Nanoparticles sizes (**A**), polydispersity (PDI) values (**B**), zeta potentials (ζ) (**C**), final loading capacities (LC) (**D**), and encapsulation efficiencies (EE) (**E**) of PLGA(BRP-187) particles prepared with an initial polymer concentration of 5, 15, and 25 mg mL^−1^ and 3% (*w*/*w* PLGA) of initial drug loading. (**F–H**) SEM images of PLGA(BRP-187) particles prepared with 5 mg mL^−1^ (P5, **F**), 15 mg mL^−1^ (P13, **G**), and 25 mg mL^−1^ (P14, **H**).

It is known for nanoprecipitation that the initial polymer concentration influences directly the final particle characteristics. With increasing polymer concentration, the particles usually become larger, and more drug molecules can be entrapped within the matrix [17,26,52]. Thus, to improve the final drug loading within the PLGA NPs, the formulation was carried out with increased initial polymer concentrations of 15 and 25 mg mL^−1^ (P13 and P14), whereas all other material parameters were fixed (BRP-187 = 3% (*w*/*w* PLGA), PVA = 0.3% (*w*/*w*), and flow rate = 2.0:0.5 mL min^−1^). The formulation of the 15 mg mL^−1^ was possible without any noticeable problems, but the 25 mg mL^−1^ concentrated solution led to increased clogging of the chip and, in consequence, to the occasional interruption and repetition of the experiment. As results of the increased polymer concentration, the particle size was increasing from d = 172 nm (P5, c = 5 mg mL^−1^) to 211 nm (P13, c = 15 mg mL^−1^) up to a size of 233 nm for P14 with c = 25 mg mL^−1^ (Figure 4A). This increase in the particle size with increasing polymer concentration was expected and is in agreement with the literature [23]. But despite the high polymer concentration, the PDI values remained low with 0.14 and 0.17 (Figure 4B), which was attributed to the rapid formulation through the chaotic mixing channel.

The zeta potential remained in the same region with −30.37 mV for P5, −34.12 mV for P13, and −32.42 mV for P14, which indicates high stability of the suspensions due to the highly negative value. With a higher concentration of PLGA, not only the particle sizes were increasing, but also the LC values increased very efficiently up to 3.3% for P14 (Figure 4D). The EE (%) was calculated to be 76% (P13) and 108% (P14), whereby the data above 100% was interpreted as complete drug encapsulation along with a minor loss of PLGA material during the purification process (Figure 4E). In order to check if the drug was really encapsulated and not partially present as free drug crystal, intensive SEM analysis was performed. The images revealed spherical and narrowly distributed particles for all polymer concentrations (Figure 4F–H). Size calculations from SEM images showed increasing particle sizes along with the increasing polymer concentration with d_SEM_ = 85, 107, and 124 nm for P5, P13, and P14, with PDI values of 0.08, 0.16, and 0.24, respectively. 

The results demonstrate that the formulation of optimized PLGA(BRP-187) NPs is possible, using the staggered herringbone chip and optimized protocol. This utilizes the flow rate ratio used, the flow velocities, as well as PVA, drug, and PLGA concentration, and leads to highest EE values along with increased LC values but similar size distributions and still narrow PDI values (<0.2). The polymer concentration has thereby the greatest influence on the successful encapsulation of the drug, which was also shown for other drug-loaded NP systems before [53,54].

### 3.4. The Crux with the Increasing Drug Feed

After reaching high drug encapsulation with an initial BRP-187 content of 3% (*w*/*w* PLGA), the starting drug feed was increased to 5 and 10% BRP-187 (*w*/*w* PLGA) to investigate the maximum BRP-187 loading while maintaining the excellent particle characteristics and stability of the suspension. The polymer concentration was set to c = 15 mg mL^−1^ to avoid experimental termination since a polymer concentration of 25 mg mL^−1^ with 3% BRP-187 (*w*/*w* PLGA) reached already the limit of possible material input without undesired side effects, such as chip clogging. As solvents, acetone (P15 and P16), and the solvent mixture Ac:THF were tested (P17 and P18). For the 5% BRP-187 (*w*/*w* PLGA) formulation in acetone, NP sizes of 214 nm were obtained (P15, Table 1). The average size increased slightly to 222 nm if 10% BRP-187 (*w*/*w* PLGA) was applied (P16). For the solvent mixture Ac:THF, sizes of 220 nm (5% BRP-187 (*w*/*w* PLGA) P17) and 237 nm (10% BRP-187 (*w*/*w* PLGA), P18) were measured. As observed before, the PDI values were rather low, between 0.13 and 0.20, in view of the high polymer concentration used for the formulations. However, small aggregation peaks were detected in the intensity distribution of the DLS, indicating the presence of aggregates or drug crystals (Table SI 2, P15–P16, in the Supplementary Materials). Subsequent in-depth analysis via SEM revealed the presence of many drug crystals for all formulations with 5 and 10% BRP-187 (*w*/*w* PLGA) next to the defined particles with spherical shape (P15–P18, Figure SI 5). One obvious reason for the observed drug crystallization is the increased initial drug concentration in the formulation. Although enough polymer material should be around the drug in the solvent due to the high polymer concentration (15 mg mL^−1^), it seems that the ratio of polymer to BRP-187 was not adequate and, therefore, led to insufficient encapsulation and precipitation/crystal formation of the drug in the nanometer range (>500 to 1000 nm) at this high concentration. Thus, during the centrifugation process, the free BRP-187 was not washed out but sedimented instead with the NPs. BRP-187 is a very hydrophobic drug, but PLGA is not a highly hydrophobic polymer [9,52]. Hence, during the solvent switch, it might be possible that the drug crystallizes much faster before it can even be surrounded by the PLGA matrix. It is also possible that the drug molecule diffuses out of the particle to the particle surface and then precipitates out in the water phase with growing crystals. Due to the high drug crystal formation, the obtained LC values of 3.80% (P15), 7.29% (P16), 3.92% (P17), and 6.86% (P18) were declared as not representative for “encapsulated” drugs, and, therefore, no EE values were calculated (Table 1).

### 3.5. Removement of Free Drug Crystals

**Figure 5:**
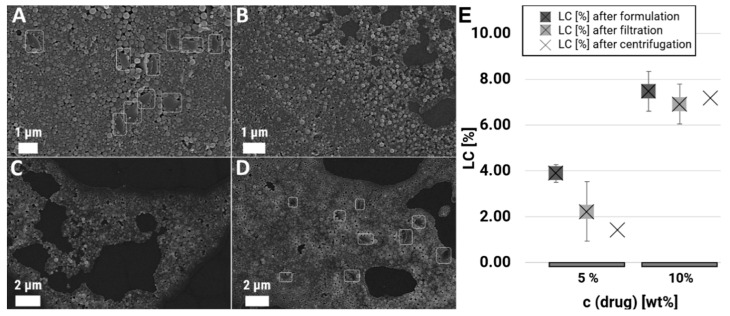
SEM images of the initial sample P15 (**A**) and after removal of the free drug crystals through centrifugation (**B**), 1st filtration (**C**), 2nd filtration (**D**). Influence of the purification method on the loading capacities for P15 and P16 with 5% and 10% (*w*/*w*) drug feed (**E**).

For the removal of the undesired drug crystals occurring during the formulations that were performed with 5 and 10% BRP-187 (*w*/*w* PLGA), two different purification procedures were tested: (i) aggregates were removed through quick centrifugation for 10 min at 4000 rpm (Figure 5B) and (ii) purification via filtration, whereby 0.45 µm (Figure 5C) and 0.80 µm (Figure 5D) size cut-off filters were tested. The purification of the samples P15–P18 by centrifugation resulted in the efficient removal of the free drug crystals (P15, Figure 5) but also in a high loss of material since not only drug crystals but also larger particles are collected within the sediment and get lost as soon as the pellet is separated from the supernatant. The final NP concentrations decreased by up to 60% (P15–18b, Table 2). Although filtration through the 0.45 µm filter could separate the drug crystals efficiently (Figure 5C), it was also accompanied by a serious loss of material in the range of 50 to 90% (Table 2). The filtration through a 0.80 µm syringe filter showed only a slight reduction of free drug crystals, and, therefore, no final drug loading values were determined for these samples. It was observed that with filtration, more material was lost in comparison to the quick centrifugation approach. The final LC values of the purified 5% BRP-187 (*w*/*w* PLGA) samples (P15 and P17) were low with 2.23% and 2.75% for the filtered suspensions and 1.43% and 1.33% for the centrifuged samples (Figure 5E, Table 2), which makes the herein applied 5% approach redundant. The 10% BRP-187 (*w*/*w* PLGA) samples P16 and P18, however, revealed still high LC values of 6.91% and 6.61% for the filtered samples as well as 7.18% and 3.95% for the centrifuged suspensions. The obtained LC values were almost three times higher than the values achieved before [9] and could be considered as high drug loads compared to the literature values where LC values above 5% have been described rarely [55].

### 3.6. Variation of the Surfactant Concentration

The surfactant is a key formulation parameter that influences the stability of the particles, prevents fast degradation of the polymer matrix, and supports the drug protection inside the particle [56,57,58]. For the formulation of polymeric NPs, a variety of surfactants, such as polysorbates, poloxamers, or PVAs, are available [59,60], whereby increased stability of drug-loaded PLGA particles produced with PVA was observed [36]. Thus, PVA was selected as a surfactant for the preparation of PLGA(BRP-187) particles [9,36]. The previous protocols with 0.3% (*w*/*w*) of PVA seem advantageous due to the low demand for surfactant. However, the so far tested protocols have revealed that the LC of BRP-187 into PLGA is just moderate at 3% since only 3% BRP-187 (*w*/*w* PLGA) can be used for stable NP formation. Higher LC values could be achieved by applying 10% BRP-187 (*w*/*w* PLGA), but then increased drug crystal formation occurred and required additional purification steps that were accompanied by high material loss. As both options are not very satisfactory, the optimization of the formulation with regard to the prevention of crystallization of the drug is clearly necessary. Successful methods to suppress drug crystallization, which have previously been described in the literature, use different surfactants, additives, or higher surfactant concentrations [58]. Since the conditions should be kept similar to the previous study [9], it was not intended to use another surfactant or add any other additives. Thus, the surfactant concentration was increased to 1% (P19) and 3% of PVA (P20) for the formulation with 15 mg mL^−1^ PLGA and 10% BRP-187 (*w*/*w* PLGA) in order to prevent drug crystal formation and to stabilize the NP suspension. The resulting particle sizes were in the same range as before with 0.3% (*w*/*w*) PVA with 220–230 nm as average diameter, but improved PDI values were observed with 0.095 to 0.161, which indicated less aggregation of the NPs and an increased stability effect of the PVA on the suspension. Unfortunately, subsequent SEM analysis revealed that the 1% (*w*/*w*) PVA concentration was not sufficient enough to suppress the formation of drug crystals in a reproducible manner as still lots of drug crystals were observed in every second formulation (P19, Figure SI 5). The LC value was calculated to be 7.0% (Table 1) but is not representative due to the presence of the drug crystals. With 3% PVA, no free drug crystals could be detected during extensive SEM analysis, showing that the drug crystal formation could be prevented with higher surfactant concentration. However, the LC value of the formulation P20 was significantly lower with LC = 3.43% and, thus, not optimized in terms of drug loading. The reduced EE with increasing surfactant can be explained with a solubilizing effect of the BRP-187 by the PVA molecules, which was also shown for other drug molecules and high surfactant concentrations [36]. Due to this enhanced solubilization of the drug within the suspension, the drug is removed from the supernatant during the centrifugation procedure.

## 4. Conclusions

With the application of the herringbone mixer chip, defined PLGA(BRP-187) particles could be prepared in size range from 120 to 260 nm with low dispersity (PDI values from 0.05 to 0.2). The final LC values and particle characteristics could be fine-tuned and improved with an optimized formulation and purification protocol. Due to the rapid mixing in the herringbone mixer chip, PLGA concentrations above 15 mg mL^−1^, as well as high PVA concentrations up to 3% (*w*/*w*), could be applied without negative effects on the size distribution of the particles (PDI < 0.2). Full encapsulation was reached with 3% BRP-187 (*w*/*w* PLGA), 0.3% (*w*/*w*) PVA as surfactant, and a polymer concentration of 25 mg mL^−1^_._ The highest drug loading with LC = 7.3% was observed for the formulation of PLGA with c = 15 mg mL^−1^ and 10% BRP-187 (*w*/*w* PLGA) with 0.3% (*w*/*w*) PVA. However, SEM images revealed in the latter formulation the formation of drug crystals, which required additional purification steps. Filtration through 0.45 μm syringe filters could successfully remove the drug crystals, but the procedure was accompanied by high material loss (up to 90%). The alternative purification approach via centrifugation was more successful and resulted in a minor loss of material (up to 60%). The LC values for the 10% BRP-187 (*w*/*w* PLGA) samples prepared with 0.3% (*w*/*w*) PVA stayed high with 6.9% and 7.2%. Increasing the PVA concentration to 1% (*w*/*w*) could not sufficiently reduce the drug crystal formation. An additional increase of the PVA concentration up to 3% (*w*/*w*) could suppress the BRP-187 drug crystallization but led to decreased LC and EE values due to enhanced solubilization of the drug within the supernatant. The herein obtained results clearly demonstrate that the BRP-187 drug loading into PLGA particles is limited and dependent in a sensitive manner on the initial concentrations of drug, polymer, and surfactant. 

This study confirmed the importance of detailed characterization of formulations using orthogonal techniques, such as DLS and SEM, to ensure efficient drug inclusion and reliable results. DLS measurements did not always reveal the prominent drug crystals, and only SEM enabled their detection. Thus, if the particles would have been only checked by DLS and UV-Vis, false-positive results would be obtained, i.e., high drug concentrations would be calculated, which are misleading since they are not entrapped within the polymer matrix and are present next to the polymer particles in the form of free drug crystals. This study also revealed that at least three independent experiments were necessary to gain a full impression if the formulation was successful or not, in particular with high drug concentrations (5 and 10% BRP-187 (*w*/*w* PLGA)). In some formulations, stable and well-defined drug-loaded NPs were observed, whereas, in other formulations prepared with the same parameters, many drug crystals were present. Thus, only repeating experiments and detailed analysis of every single formulation can provide the answer if a formulation protocol is reliable or would need further improvement. 

Through the development of an optimized protocol for the formulation of BRP-encapsulating PLGA particles, not only the change from the classic batch to an easily controllable microfluidic formulation has been achieved but also a significant increase in the LC, which is one of the main challenges in the development of drug delivery systems. Due to the large number of auspicious hydrophobic drugs that are already on the market but still lack bioavailability and efficient encapsulation, there is a wealth of applications for microfluidics and the protocols developed here. The established protocols can be used as a starting point for the formulation of new drug-loaded PLGA particles as well as expanded to other polymer systems and surfactants. In addition, this study will help to further develop stable (BRP-187) formulations so that the active substance can be effectively encapsulated and delivered to its targeted site of action in sufficient doses.

## Figures and Tables

**Table 1:** PLGA(BRP-187) particle characteristics after formulation using different flow rates, solvents, PVA contents, initial polymer, and drug concentrations.

		Formulation Parameter				DLS/ELS Measurement				Drug Content	
P#	Solvent	BRP-187 % (*w*/*w*)	c_PLGA_ [mg mL^−1^]	c_PVA_ [%]	^a^ Q_W_/Q_PS_ [mL min^−1^]		^b^ Z_avg_ [nm]	PDI	^c^ ζ [mV]	^d^ LC [%]	^d^ EE [%]
P1	Ac	0	5	0.3	0.4:0.1		194	0.11	−32.80	-	-
P2	Ac	0	5	0.3	2.0:0.5		175	0.05	−30.00	-	-
P3	Ac	0	5	0.3	8.0:2.0		119	0.18	−30.20	-	-
P4	Ac	3	5	0.3	0.4:0.1		212	0.09	−30.90	0.69	23.03
P5	Ac	3	5	0.3	2.0:0.5		172	0.05	−30.37	0.42	14.10
P6	Ac	3	5	0.3	4.0:0.5		137	0.07	−23.70	0.33	10.91
P7	Ac	3	5	0.3	6.0:0.5		141	0.10	−27.70	0.59	19.57
P8	Ac	3	5	0.3	8.0:0.5		138	0.16	−27.20	0.89	29.58
P9	Ac	3	5	0.3	8.0:2.0		121	0.12	−33.25	0.59	19.80
P10	^e^ Ac:THF	3	5	0.3	2.0:0.5		194	0.10	−34.40	0.89	29.82
P11	^f^ Ac:EtOAc	3	5	0.3	2.0:0.5		246	0.09	−34.10	0.45	15.10
P12	ACN	3	5	0.3	2.0:0.5		196	0.10	−29.10	0.59	19.51
P13	Ac	3	15	0.3	2.0:0.5		211	0.14	−34.12	2.29	76.35
P14	Ac	3	25	0.3	2.0:0.5		233	0.17	−32.42	3.25	108.31
P15	Ac	5	15	0.3	2.0:0.5		214	0.18	−29.17	3.80 *	*
P16	Ac	10	15	0.3	2.0:0.5		222	0.17	−29.02	7.29 *	*
P17	^e^ Ac:THF	5	15	0.3	2.0:0.5		220	0.13	−27.00	3.92 *	*
P18	^e^ Ac:THF	10	15	0.3	2.0:0.5		237	0.20	−31.67	6.86 *	*
P19	Ac	10	15	1.0	2.0:0.5		259	0.18	−29.95	7.00 *	*
P20	Ac	10	15	3.0	2.0:0.5		245	0.12	−29.03	3.43	34.33

Ac = acetone, ACN = acetonitrile, THF = tetrahydrofuran, EtOAc = ethyl acetate. ^a^ Q_w_:Q_PS_ flow rate ratio of water phase (0.3%/1.0%/3.0% (*w*/*w*) PVA) to polymer solution. ^b^ DLS measurements carried out with 1:10 dilution with Milli Q water, 5 measurements a 30 s. ^c^ Zeta potential measured with 1:100 dilution in Milli Q. ^d^ Drug concentration determined via UV-Vis in DMSO. Drug loading capacity (LC) and encapsulation efficacy (EE) related to PLGA (without PVA residue). ^e^ Ac:THF ratio 3:1. ^f^ Ac:EtOAc ratio 3:1. * Due to the high drug crystal formation, the LC is not representative for “encapsulated” drugs, and, therefore, no EE values were calculated for these samples.

**Table 2:** PLGA(BRP-187) particle characteristics before and after additional purification.

P#	Solvent	BRP-187 % (*w*/*w*)	c_PLGA_ [mg mL^−1^]	c_NP_ [mg mL^−1^]	^a^ LC [%]	^a^ EE [%]
**P15**	Ac	5	15	8.20	3.80	*
**^f^** **P15_f**				0.62	2.23	44.60
**^c^** **P15_c**				3.13	1.43	28.57
**P16**	Ac	10	15	8.38	7.29	*
**^f^** **P16_f**				1.08	6.91	69.14
**^c^** **P16_c**				3.19	7.18	71.79
**P17**	^b^ Ac/THF	5	15	5.00	3.92	*
**^f^** **P17_f**				1.97	2.75	54.96
**^c^** **P17_c**				2.30	1.33	26.61
**P18**	^b^ Ac/THF	10	15	5.60	6.86	*
**^f^** **P18_f**				2.30	6.61	66.12
**^c^** **P18_c**				2.42	3.95	39.46

Ac = acetone, THF = tetrahydrofuran. ^a^ Drug concentration determined via UV-Vis in DMSO. Drug loading capacity (LC) and encapsulation efficacy (EE) related to PLGA (without PVA residue). ^b^ Ac/THF ratio 3:1. ^f^ Purified by filtration (0.45 μm syringe filter). ^c^ Purified by centrifugation (10 min at 4000 rpm). * Due to the high drug crystal formation, the LC is not representative for “encapsulated” drugs, and, therefore, no EE values were calculated for these samples.

## Supplementary Materials

The following are available online at https://www.mdpi.com/2073-4360/12/11/2751/s1. Figure SI 1. Synthesis of BRP-187 according to Banoglu. Analysis was performed via ^1^H NMR spectroscopy and mass spectrometry; Figure SI 2. Workflow of PLGA(BRP-187) particles formulation. Figure SI 3. (A) Size and PDI values of empty and BRP-187 loaded PLGA NPs: Formulation done without drug (P1-P3) and with BRP-187 feed of 3% (*w*/*w*) PLGA) (P4, P5, P8) applying different flow rate velocities and ratios. (B) Comparison of SEM images for empty and drug loaded NP at two different flow rates (Q_w_:Q_PS_ 2.0:0.5 and 8.0:2.0); Figure SI 4. (A) Size and PDI values of the particles formulated with different flow rate ratios and flow rate velocities applying a PVA concentration of 0.3% (*w*/*w*) (P4-P9). (B) LC values of the particles P4-P9; Figure SI 5. EM image of the particles prepared with 3%, 5% and 10% (*w*/*w*) BRP-187 drug feed using 15 mg mL-1 PLGA in acetone (P13, P15, P16) or acetone/THF (P17, P18) and 10% (*w*/*w*) BRP-187 drug feed using 1% PVA (P24) and 3% (*w*/*w*) PVA (P25). All suspensions were imaged after purification, P15-18 were further filtered through a 0.45 um syringe filter in order to purify the particles from the free drug crystals; Figure SI 6. BRP-187 calibration curve for the calculation of the LC and EE. Table SI 1. Overview of all formulations and the resulting particle characteristics; Table SI 2. DLS intensity plots of single formulations after purification step.

## Abbreviations

Ac	Acetone
ACN	Acetonitrile
DMSO	Dimethyl sulfoxide
EE	Encapsulation efficiency
EtOAc	Ethyl acetate
FLAP	5-lipoxygenase-activating protein
GA	Glycolic acid
LA	Lactic acid
LO	lipoxygenase
LC	Loading capacity
mPGES-1	Microsomal prostaglandin E_2_ synthase-1
NSAIDs	Non-steroidal anti-inflammatory drugs
NPs	Nanoparticles
PLGA	Poly(lactic-*co*-glycolic acid)
PTFE	Polytetrafluoroethylene
PVA	Poly(vinyl alcohol)
PGE2	Prostaglandin E2
SEM	Scanning electron microscopy
THF	Tetrahydrofuran
