# Supplementary material for: Optimized Encapsulation of the FLAP/PGES-1 Inhibitor BRP-187 in PVA-Stabilized PLGA Nanoparticles Using Microfluidics
Source: Polymers (Basel). 2020 Nov 20;12(11):2751. doi: 10.3390/polym12112751 (PMC7699897; doi:10.3390/polym12112751)
Supplement: Supplementary file 1 [file polymers-12-02751-s001.pdf]

Supporting information

Optimized encapsulation of the FLAP/PGES-1 inhibitor BRP-187 in PVA-stabilized PLGA nanoparticles using microfluidics

Mira Behnke[a, b, #], Antje Vollrath[a, b, #], Lea Klepsch[a], Bärbel Beringer-Siemers[a], Steffi Stumpf[a, b], Justyna A. Czaplewska[a], Stephanie Hoeppener[a, b], Oliver Werz[b, c], Ulrich S. Schubert[a, b,*]

[a]Laboratory of Organic and Macromolecular Chemistry (IOMC), Friedrich Schiller University Jena, Humboldtstraße 10, 07743 Jena, Germany
[b]Jena Center for Soft Matter (JCSM), Friedrich Schiller University Jena, Philosophenweg 7, 07743 Jena, Germany
[c] Department of Pharmaceutical/Medicinal Chemistry, Institute of Pharmacy, Friedrich Schiller University Jena, Philosophenweg 14, 07743, Jena, Germany

* Corresponding author
# shared first author

1. Drug synthesis

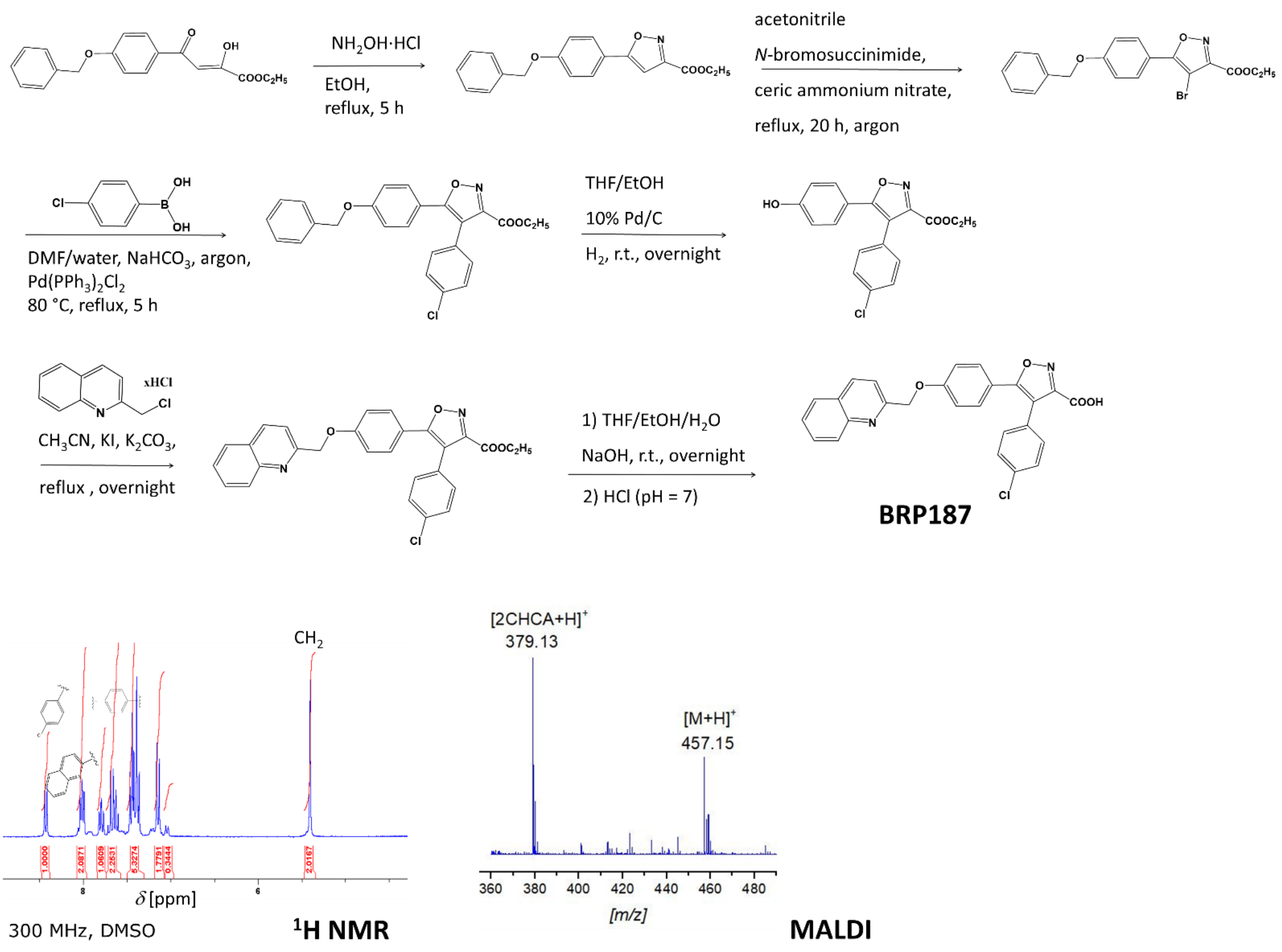

Figure SI 1. Synthesis of BRP-187 according to Banoglu [1]. Analysis was performed *via* ^{1}H NMR spectroscopy and mass spectrometry.

2. Workflow of the formulation

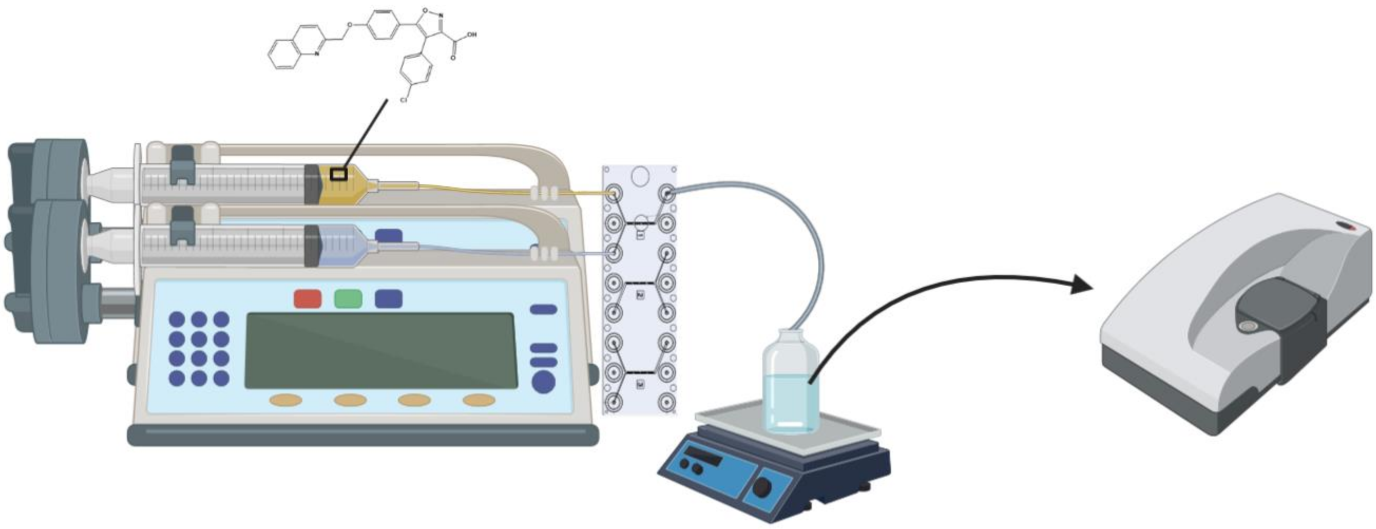

Figure SI 2. Workflow of PLGA[BRP-187] particles formulation [2].

3. Formulation overview

Table SI 1. Overview of all formulations and the resulting particle characteristics.

	**Formulation parameter**							**Nanoparticle characteristics**						**SEM Analysis**			**Drug content**			**Resuspension**	
**P#**	**Solvent**	**BRP [w/w]**	**c_{PLGA} [mg mL^{-1}]**	**PVA [w/w]**	**[a]Q_W/Q_{PS} [mL min^{-1}]**	**V_w [mL]**	**V_{PS} [mL]**	**[b]Z_{avg} [nm]**	**PDI**	**[c]ζ [mV]**	**c_{NP} [mg mL^{-1}]**	**c_{PVA} [mg mL^{-1}]**	**Yield**	**Size [nm]**	**SD**	**PDI**	**c_{BRP} [µg mL^{-1}]**	**[d]LC%**	**[d]EE%**	**Z_{avg} [nm]**	**PDI**
P1	Ac	-	5	0.3	0.4:0.1	4	0.5	194	0.11	-32.80	5.50	0.089	86.57	97	27.45	0.08	-	-	-	733*	0.68
P2	Ac	-	5	0.3	2.0:0.5	8	1	175	0.05	-30.00	9.61	0.102	76.06	88	39.95	0.18	-	-	-	437*	0.46
P3	Ac	-	5	0.3	8.0:2.0	8	1	119	0.18	-30.20	1.15	0.995	1.55	73	20.78	0.06	-	-	-	506	0.47
P4	Ac	3	5	0.3	0.4:0.1	4	0.5	212	0.09	-30.90	4.89	0.096	76.71	119	44.45	0.17	33.13	0.69	23.03	736*	0.63
P5	Ac	3	5	0.3	2.0:0.5	8	1	172	0.05	-30.37	7.76	0.140	76.16	85	26.67	0.08	32.23	0.42	14.10	281*	0.14
P6	Ac	3	5	0.3	4.0:0.5	12	1	137	0.07	-23.70	4.59	0.108	71.71	73	25.27	0.09	14.67	0.33	10.91	226	0.11
P7	Ac	3	5	0.3	6.0:0.5	16	1	141	0.10	-27.70	2.66	0.141	40.30	68	21.69	0.07	14.79	0.59	19.57	252	0.13
P8	Ac	3	5	0.3	8.0:0.5	17	1	138	0.16	-27.20	1.08	0.267	15.85	130	65.09	0.33	7.21	0.89	29.58	251	0.18
P9	Ac	3	5	0.3	8.0:2.0	8	1	121	0.12	-33.25	3.03	0.300	27.30	81	20.80	0.05	16.22	0.59	19.80	184*	0.15
P10	[e]Ac/THF	3	5	0.3	2.0:0.5	8	1	194	0.10	-34.40	5.91	0.097	58.13	86	21.58	0.05	52.00	0.89	29.82	210*	0.07
P11	[f]Ac/EtOAc	3	5	0.3	2.0:0.5	8	1	246	0.09	-34.10	4.09	0.080	40.10	121	30.85	0.08	18.17	0.45	15.10	286*	0.23
P12	ACN	3	5	0.3	2.0:0.5	8	1	196	0.10	-29.10	6.33	0.093	62.37	112	46.40	0.19	36.51	0.59	19.51	279*	0.23
P13	Ac	3	15	0.3	2.0:0.5	4	0.5	211	0.14	-34.12	9.38	0.081	61.96	107	41.09	0.16	212.89	2.29	76.35	295*	0.18
P14	Ac	3	25	0.3	2.0:0.5	4	0.5	233	0.17	-32.42	10.23	0.069	40.65	124	51.41	0.24	330.21	3.25	108.31	256*	0.16
P15	Ac	5	15	0.3	2.0:0.5	4	0.5	214	0.18	-29.17	8.28	0.080	54.67	115	48.98	0.21	311.20	3.80	75.91	223*	0.15
[g]P15_f	Ac	5	15	0.3	2.0:0.5	4	0.5	-	-	-	0.70	0.080	-	-	-	-	13.81	2.23	44.60	-	-
[h]P15_c	Ac	5	15	0.3	2.0:0.5	4	0.5	-	-	-	3.21	0.080	-	-	-	-	44.66	1.43	28.57	-	-
P16	Ac	10	15	0.3	2.0:0.5	4	0.5	222	0.17	-29.02	8.46	0.078	55.88	114	48.21	0.22	610.88	7.29	72.87	232*	0.18
[g]P16_f	Ac	10	15	0.3	2.0:0.5	4	0.5	-	-	-	1.16	0.078	-	-	-	-	74.87	6.91	69.14	-	-
[h]P16_c	Ac	10	15	0.3	2.0:0.5	4	0.5	-	-	-	3.27	0.078	-	-	-	-	229.35	7.18	71.79	-	-
P17	[e]Ac/THF	5	15	0.3	2.0:0.5	4	0.5	220	0.13	-27.00	5.07	0.076	33.31	106	45.82	0.20	196.07	3.92	78.47	239*	0.14
[g]P17_f	[e]Ac/THF	5	15	0.3	2.0:0.5	4	0.5	-	-	-	2.04	0.076	-	-	-	-	54.08	2.75	54.96	-	-
[h]P17_c	[e]Ac/THF	5	15	0.3	2.0:0.5	4	0.5	-	-	-	2.37	0.076	-	-	-	-	30.55	1.33	26.61	-	-
P18	[e]Ac/THF	10	15	0.3	2.0:0.5	4	0.5	237	0.19	-31.67	5.68	0.076	37.36	108	53.08	0.26	384.26	6.86	68.56	258*	0.20
[g]P18_f	[e]Ac/THF	10	15	0.3	2.0:0.5	4	0.5	-	-	-	2.38	0.078	-	-	-	-	152.31	6.61	66.12	-	-
[h]P18_c	[e]Ac/THF	10	15	0.3	2.0:0.5	4	0.5	-	-	-	2.50	0.078	-	-	-	-	95.55	3.95	39.46	-	-
P19	Ac	10	15	1.0	2.0:0.5	4	0.5	259	0.18	-29.95	5.59	0.144	36.30	123	59.26	0.293	381.33	7.00	70.03	228*	0.16
P20	Ac	10	15	3.0	2.0:0.5	4	0.5	245	0.12	-29.03	6.93	0.234	44.64	87	58.90	0.403	229.92	3.43	34.33	245*	0.19

Ac = acetone, ACN = acetonitrile, THF = tetrahydrofuran, EtOAc = ethyl acetate. [a] Q_w/Q_{PS} flow rate ratio of water phase (0.3%/1.0/3.0% [w/w] PVA) to polymer solution. [b] DLS measurements carried out with 1:10 dilution with milli Q water, 5 measurements a 30 sec. [c] Zeta potential measured with 1:100 dilution in milli Q. [d] Drug concentration determined *via* UV/Vis in DMSO. Drug loading capacity (LC) and encapsulation efficacy (EE) related to PLGA (without PVA residue). [e] Ac/THF ratio 3:1. [f] Ac/EtOAc ratio 3:1. [g]Purified by filtration, [h]Purified by centrifugation, *Addition of 0.3 (w/w) PVA before lyophilization.

4. Establishment of the method and comparison empty and loaded NP

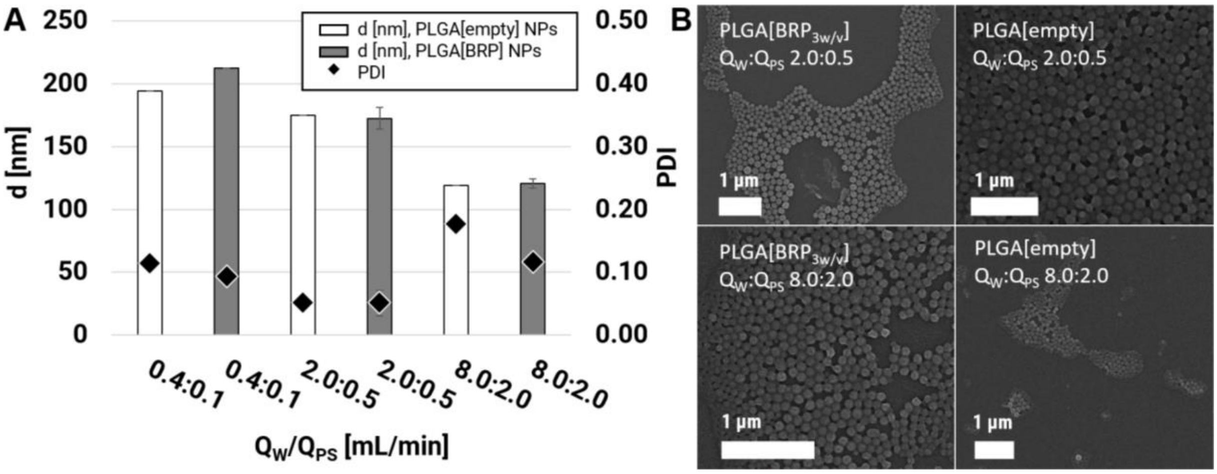

Figure SI 3. (**A**) Size and PDI values of empty and BRP-187 loaded PLGA NPs: Formulation done without drug (**P1-P3**) and with BRP-187 feed of 3% [w/w PLGA] (**P4, P5, P8**) applying different flow rate velocities and ratios. (**B**) Comparison of SEM images for empty and drug loaded NP at two different flow rates (Q_W:Q_{PS} 2.0:0.5 and 8.0:2.0)

5. Variation of the flow rates and PVA concentration

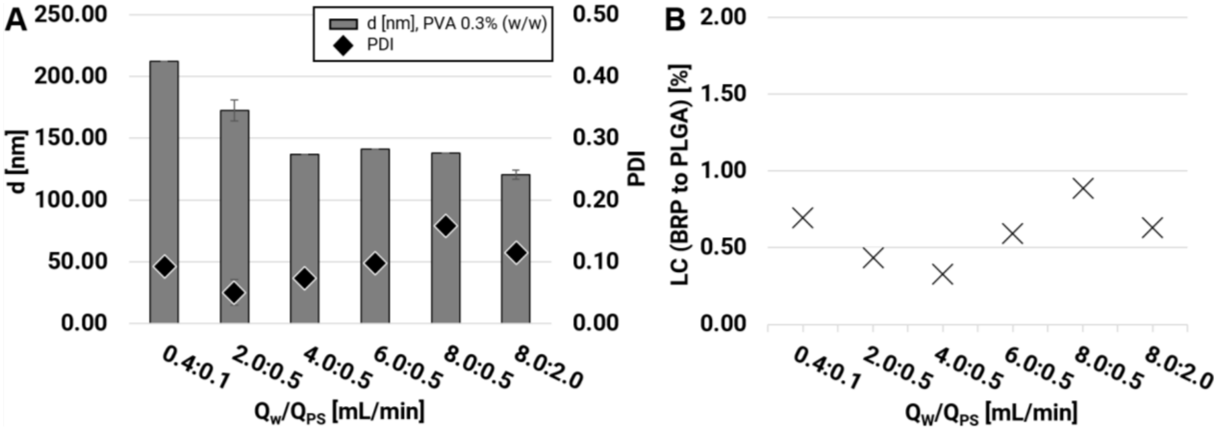

Figure SI 4. (**A**) Size and PDI values of the particles formulated with different flow rate ratios and flow rate velocities applying a PVA concentration of 0.3% [w/w] (**P4-P9**). (**B**) LC values of the particles **P4-P9**.

6. Influence of initial polymer concentration and drug feed

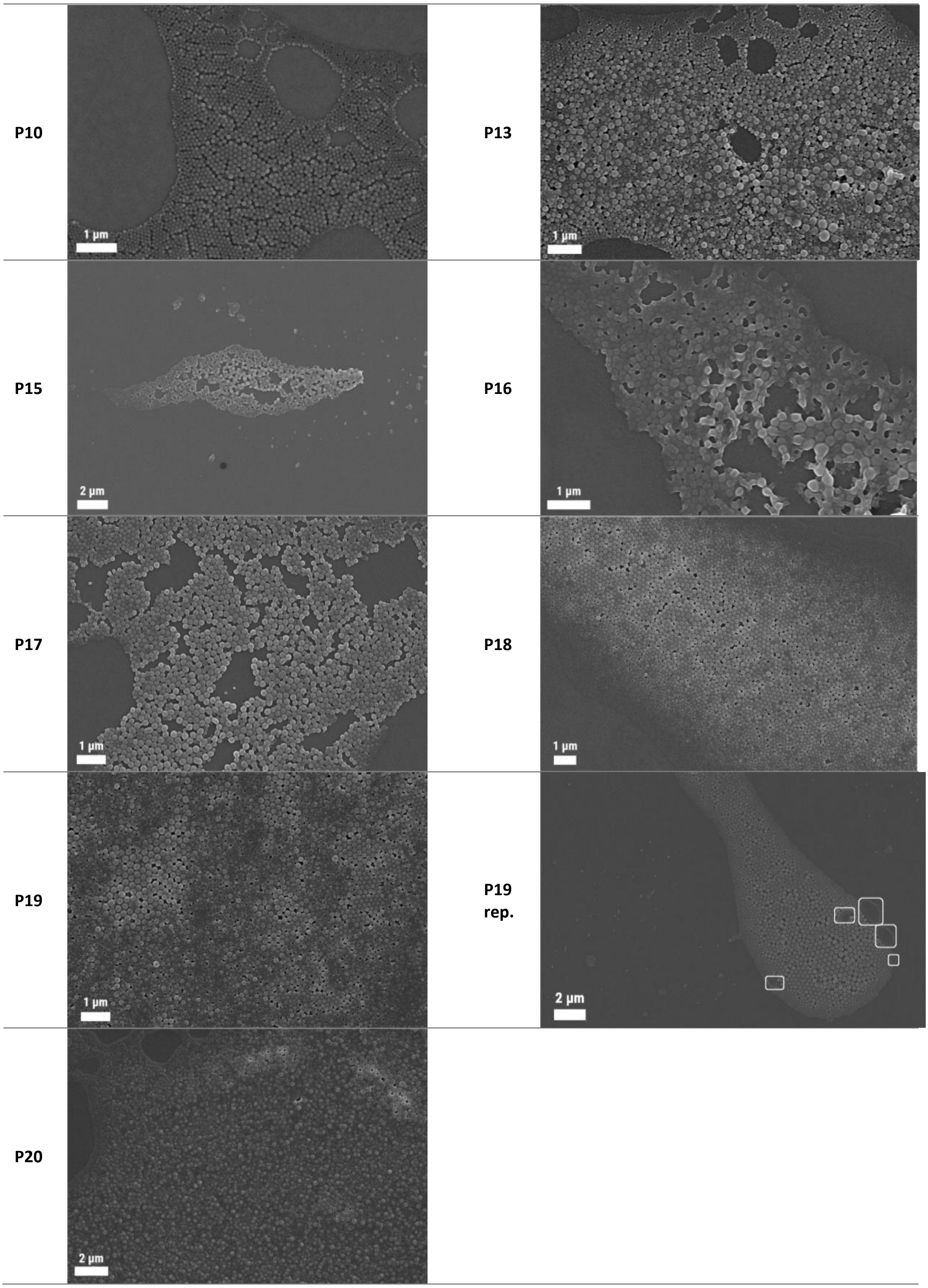

Figure SI 5. SEM image of the particles prepared with 3%, 5% and 10% [w/w] BRP-187 drug feed using 15 mg mL^{-1} PLGA in acetone (**P13, P15, P16**) or acetone/THF (**P17, P18**) and 10% [w/w] BRP-187 drug feed using 1% PVA (P24) and 3% [w/w] PVA (P25). All suspensions were imaged after purification, **P15-18** were further filtered through a 0.45 um syringe filter in order to purify the particles from the free drug crystals.

7. DLS size distribution curves of all formulations

SI Table 2. DLS intensity plots of single formulations after purification step.

P1	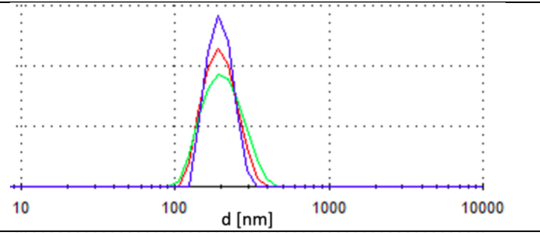	P2	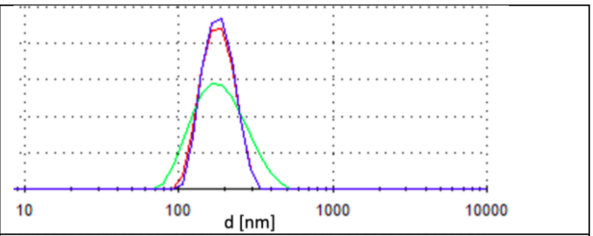
P3	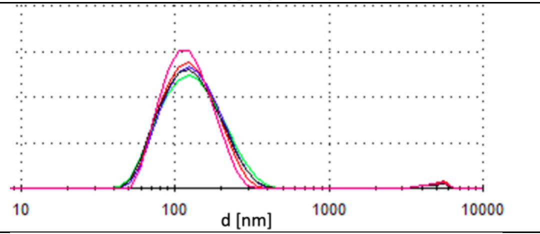	P4	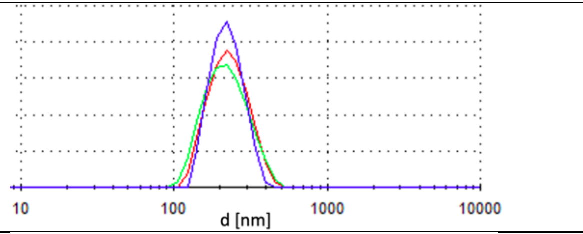
P5a	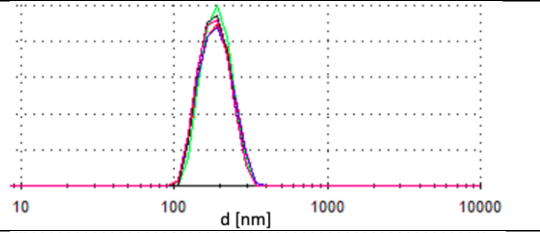	P5b	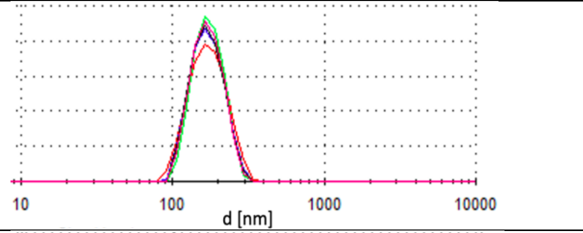
P5c	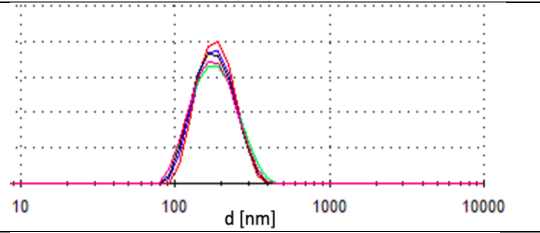	P6	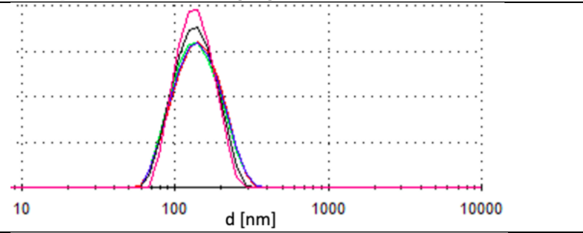
P7	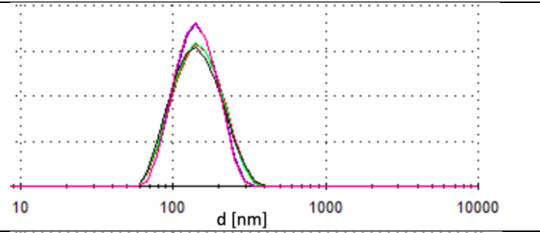	P8	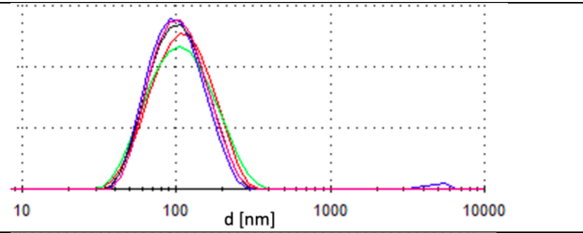
P9a	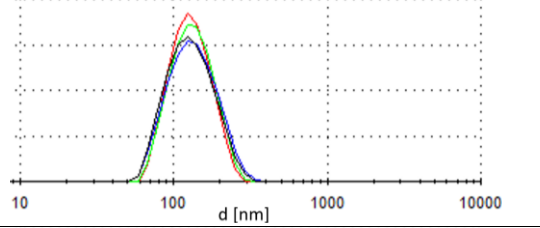	P9b	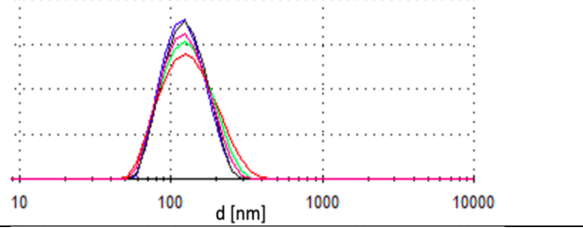
P10	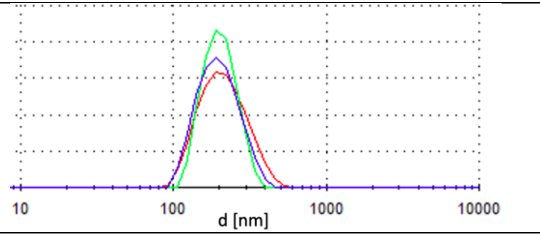	P11	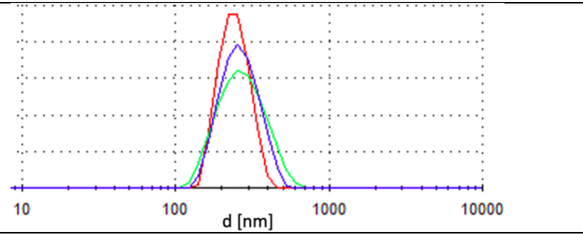

P12

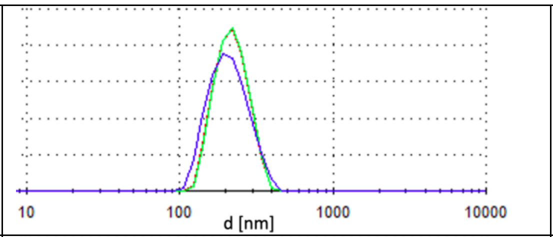

P13a

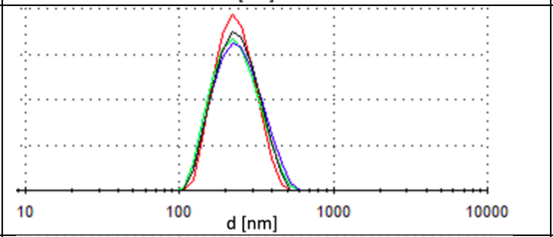

P13b

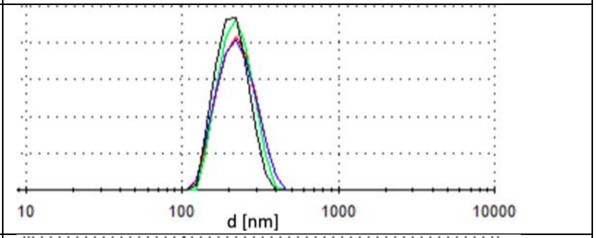

P13c

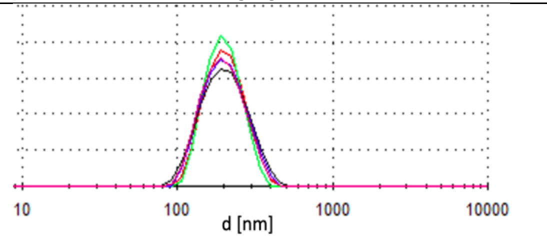

P14a

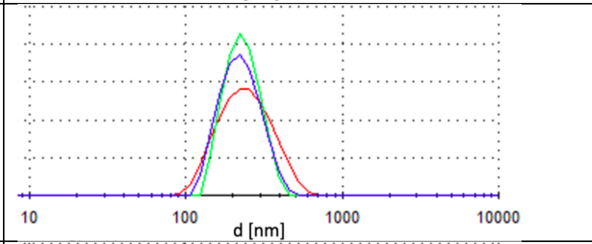

P14b

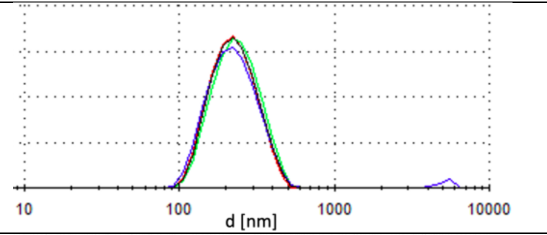

P14c

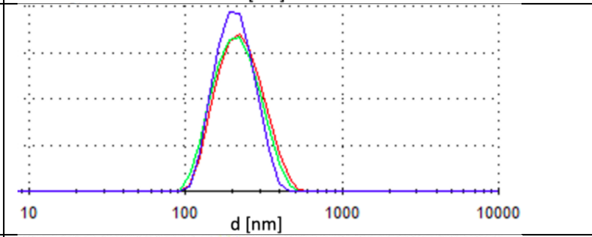

P15a

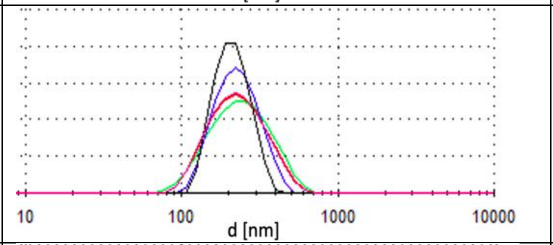

P15b

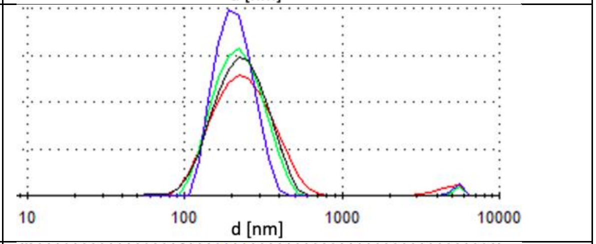

P15c

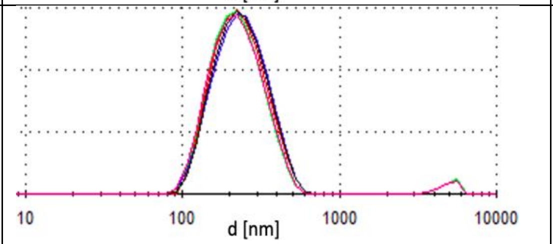

P16a

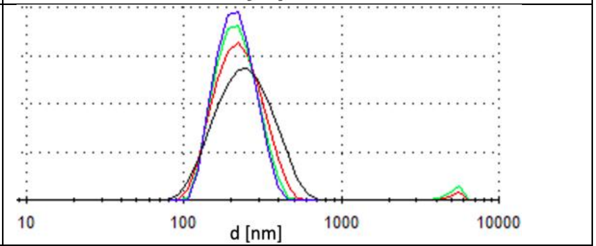

P16b

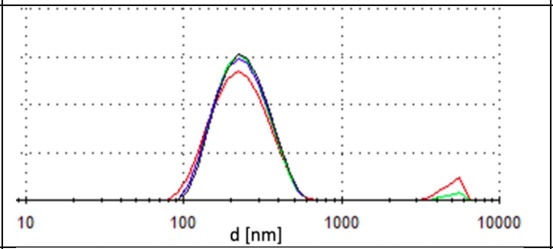

P16c

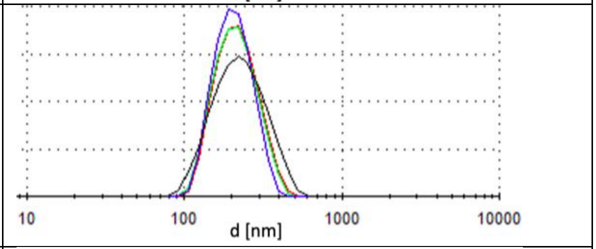

P17a

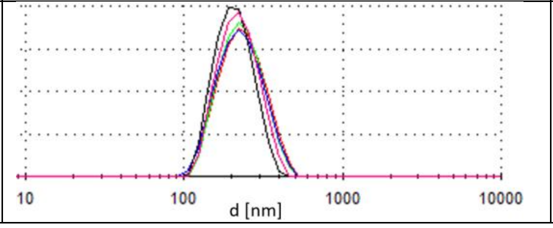

P17b

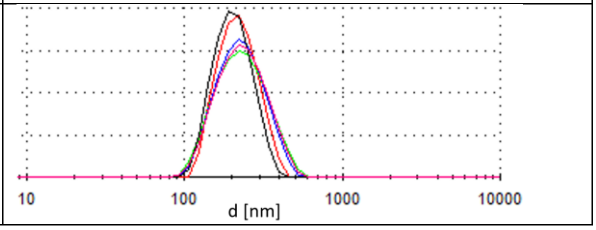

P17c	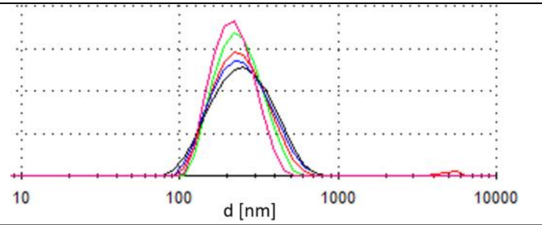	P18a	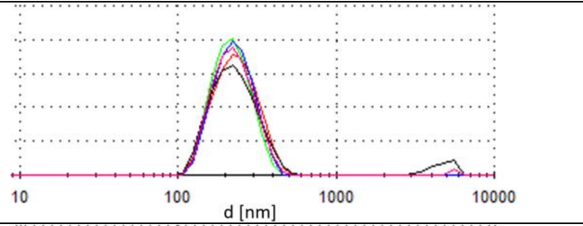
P18b	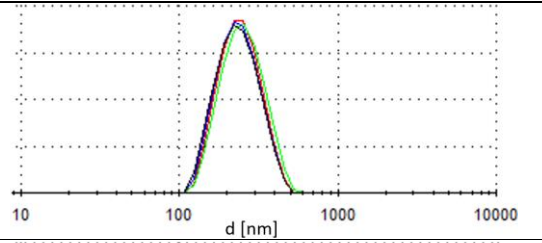	P18c	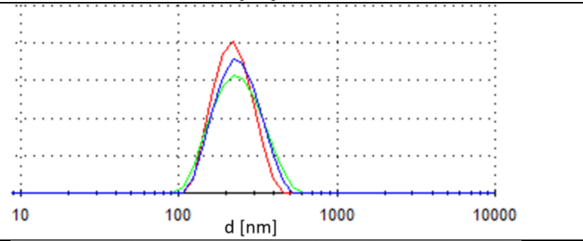
P19a	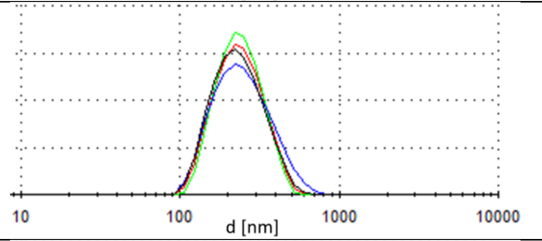	P19b	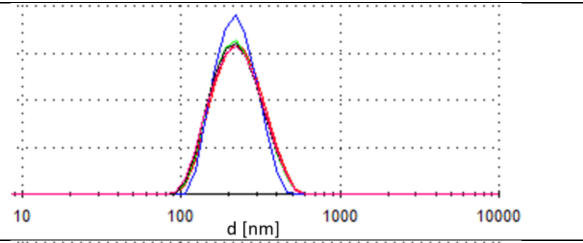
P19c	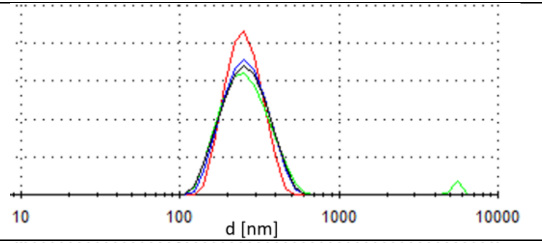	P20a	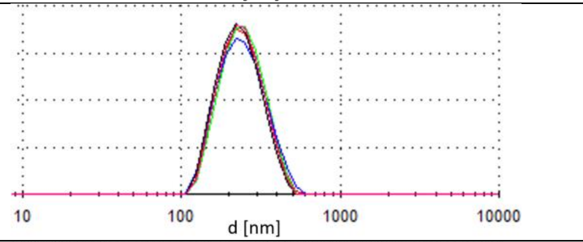
P20b	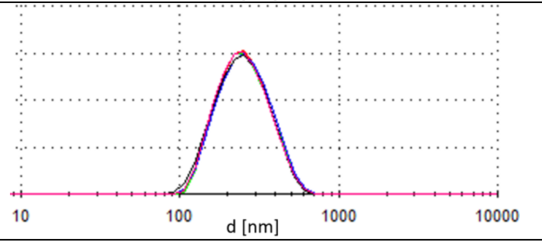	P20c	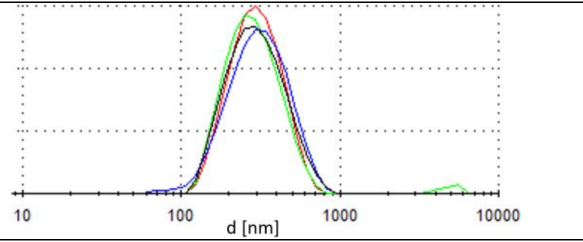

8. UV/Vis Calibration function of BRP-187

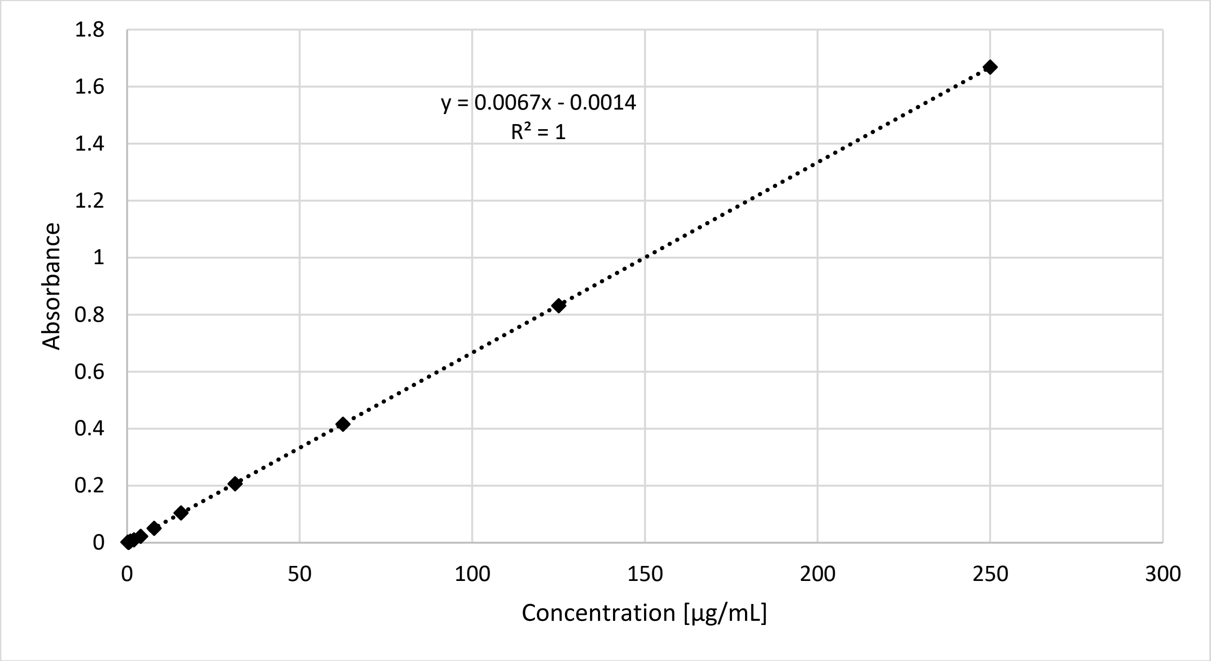

Figure SI 6. BRP-187 calibration curve for the calculation of the LC and EE.

9. SEM Analysis

For the evaluation of the NPs sizes from the SEM images ImageJ was used which provides the area of each detected particle.

$$\boldsymbol{A = \pi * r^2 \rightarrow A = \pi * \left(\frac{d}{2}\right)^2 \rightarrow d = \sqrt{\frac{A}{\pi}} * 2}$$

11. References

[1] Banoglu, E.; Celikoglu, E.; Volker, S.; Olgac, A.; Gerstmeier, J.; Garscha, U.; Caliskan, B.; Schubert, U. S.; Carotti, A.; Macchiarulo, A.; Werz, O., 4,5-Diarylisoxazol-3-carboxylic acids: A new class of leukotriene biosynthesis inhibitors potentially targeting 5-lipoxygenase-activating protein (FLAP), *Eur. J. Med. Chem.*, **2016**, 113, 1-10.
[2] C.w. BioRender.com.
